# Extract of* Sesbania grandiflora* Ameliorates Hyperglycemia in High Fat Diet-Streptozotocin Induced Experimental Diabetes Mellitus

**DOI:** 10.1155/2016/4083568

**Published:** 2016-05-22

**Authors:** Ghanshyam Panigrahi, Chhayakanta Panda, Arjun Patra

**Affiliations:** ^1^Department of Pharmacology, Royal College of Pharmacy and Health Sciences, Berhampur, Odisha 760002, India; ^2^Institute of Pharmaceutical Sciences, Guru Ghasidas Vishwavidyalaya, Bilaspur, Chhattisgarh 495009, India

## Abstract

*Background. Sesbania grandiflora* has been traditionally used as antidiabetic, antioxidant, antipyretic, and expectorant and in the management of various ailments.* Materials and Methods.* The study evaluates the antidiabetic activity of methanolic extract of* Sesbania grandiflora* (MESG) in type 2 diabetic rats induced by low dose streptozotocine and high fat diet. Diabetic rats were given vehicle, MESG (200 and 400 mg/kg, p.o.), and the standard drug, metformin (10 mg/kg), for 28 days. During the experimental period, body weight, abdominal girth, food intake, fasting serum glucose, urine analyses were measured. Insulin tolerance test was carried out on 25th day of drug treatment period. Serum analyses for lipid profile and SGOT and SGPT and serums creatinine, urea, protein, SOD, and MDA were also carried out. At the end of the experiment, animals were euthanized, the liver and pancreas were immediately dissected out, and the ratio of pancreas to body weight and hepatic glycogen were calculated.* Results.* MESG (200 and 400 mg/kg, p.o.) induced significant reduction (*P* < 0.05) of raised blood glucose levels in diabetic rats and also restored other parameters to normal level.* Conclusion.* Therefore, it is concluded that MESG has potential antihyperglycemic and antihyperlipemic activities and alleviate insulin resistance conditions.

## 1. Introduction

Diabetes mellitus is a metabolic disorder characterized by altered glucose and lipid metabolism leading to persistent hyperglycemia. High fat diets and oxidative damage may contribute to the development of diabetes mellitus which is associated with hyperglycemia, insulin resistance, dyslipidaemia, abdominal obesity, and fatty liver and is characterized by chronic polyuria, polydipsia, polyphagia, and weakness due to disturbance in carbohydrate, fat, and protein metabolism. The chronic hyperglycemia of diabetes is associated with long-term micro- and macrovascular complications such as damage, dysfunction, and failure of various organs, especially the eyes, kidneys, nerves, heart, and blood vessels [1, 2]. The increasing availability of energy dense food and the sedentary lifestyle that is becoming prevalent in both first-world and developing nations have led to a worldwide epidemic in type 2 diabetes mellitus (T2DM). Diabetes currently afflicts more than 220 million people worldwide and this will increase to about 552 million by 2030 [3–5] of which the developing countries contribute more to this increase.

A number of plants are mentioned in ancient Indian literature for the treatment of hyperglycemic and hyperlipidemic conditions. One such drug is* Sesbania grandiflora* (family Leguminosae: Papilionoideae), being used by some local tribal people, and is selected for the present study.* Sesbania grandiflora,* also called Agati, is an open branching tree up to 15 m tall and 30 cm in diameter that commonly grows on dikes between rice paddies, along roadsides, and in backyard vegetable gardens.* S. grandiflora* native range through Tropical Asia including India, Indonesia, Malaysia, Myanmar, and Philippines with possibly Indonesia as the centre of the diversity and Southeast Asia is noncontiguous. Galactomannans, oleanolic acid, *β*-sitosterol, and carbohydrates have been reported in the plant. Traditionally, the bark is used as astringent and utilized for the treatment of smallpox, ulcers in the mouth and alimentary canal, infantile disorders of stomach, and scabies; the juice of the leaves is utilized for the treatment of epileptic fits and clinical research supports the anticonvulsive activity of Agati leaves [6]. Traditionally, the plant has also been used for its astringent, bitter, thermogenic, styptic, alexeteric, anthelmintic, demulcent, constipating, expectorant, and antipyretic characteristics and in treatment of bronchitis, cough, vomiting, wounds, ulcers, diarrhoea, dysentery, internal and external haemorrhages, dental caries, oral ulcers, proctoptosis, stomatitis, and intermittent fevers. Methanolic extract of the plant has been studied for* in vitro* antioxidant activity and total phenolic and total flavonoid content [7–13]. The present study is designed to investigate the scientific basis for its traditional usage in the treatment of diabetes mellitus. The study was carried out to screen the antidiabetic activity of methanol extracts of* Sesbania grandiflora* in high fat diet and low dose streptozotocin induced type 2 diabetic rats.

## 2. Materials and Methods

### 2.1. Plant Material and Extract Preparation

The leaves of* Sesbania grandiflora* were collected from local areas of Berhampur, Odisha, India, and were identified and authenticated by Dr. P. Lakshminarasimhan, Scientist, Central National Herbarium, Botanical Survey of India, Howrah, India (authentication number CNH/23/2011/Tech.II/483). The plant materials were air-dried under shade, coarsely powdered, and kept in airtight container. Methanolic extract of* S. grandiflora* (MESG) was prepared by soxhlet apparatus by successive extraction with petroleum ether (60–80°C), chloroform, and methanol. Petroleum ether and chloroform were used in initial steps of extraction for defatting the plant materials. The methanolic extract was collected and dried using rotary vacuum evaporator followed by lyophilization and stored in desiccator until further use.

### 2.2. Phytochemical Screening

Qualitative analysis for confirming different groups of phytoconstituents in MESG was carried out based on standard protocols [14–16].

### 2.3. Animals

The Sprague-Dawley (SD) rats of both sexes used for the present work were procured from the animal house of the institute (RCPHS, Berhampur, Odisha, India) and housed in polypropylene cages with clean sterilized husk bedding (three rats/cage). Animals were maintained under controlled room temperature (22 ± 2°C) and humidity (55 ± 5°C) with a 12 : 12 hours' light : dark cycle. The animals were acclimatized to laboratory hygienic conditions for 7 days before commencing the experiment. The animals were fed with standard laboratory food diet made in-house as recommended by National Institute of Nutrition (NIN), Hyderabad, and pure drinking water* ad libitum*. The ethical clearance was granted by Institutional Animal Ethics Committee (IAEC) of Royal College of Pharmacy and Health Sciences, Berhampur, Odisha, India (registration number 1018/C/06/CPCSEA).

### 2.4. Drugs and Chemicals

Streptozotocin (STZ) was purchased from HiMedia Laboratories Pvt. Ltd., Mumbai. Metformin was obtained from Dr. Reddy's laboratories, Hyderabad. Different biochemical analysis kits were purchased from Crest Biosystems, a division of Coral Clinical Systems, India. Glucometer and blood glucose test strips of Contour TS, Bayer Pharmaceuticals Pvt. Ltd., India, and urine analysis reagent strips (glucose-ketone) of Dirui Industrial Co. Ltd., China, were purchased. All other chemicals used for the study were of analytical grade.

### 2.5. Induction of T2DM

Many studies have reported that SD rats fed with high fat diet (HFD) and injected with a low dose of STZ could serve as an alternative animal model for simulating type 2 diabetes in humans [17–19]. Similar protocol with little modification was used to induce T2DM in the animals. Briefly, the rats were allowed to feed with two dietary regimens, group one consisting of 24 rats in treatment group and group two comprising 6 rats as control. After 7 days of acclimatization, rats in treated group were allocated into dietary regimens for the initial period of 4 weeks (i.e., diet manipulating period) consisting of HFD to develop obesity induced dyslipidemia. HFD contains normal laboratory food 67.5%, Lard 31%, cholesterol 1%, Dl-methionine 0.3%, yeast powder 0.1%, and sodium chloride 0.1%. HFD consists of fat (58%), proteins (25%), and carbohydrate (17%) as percentage of total kcal. The control group was continually fed with normal laboratory food only. All rats had free access to food and water. After 4 weeks of dietary manipulation, rats were fasted for 12 h (free access to water) and injected i.p. with 35 mg/kg of STZ in 0.1 M citrate buffer, pH 4.5. In contrast, rats in the control group were injected with 0.1 M citrate buffer solution only. After administration of STZ, the treated group animals had free access to HFD feed. The control group animals were continually fed with normal laboratory food. The development of hyperglycemic condition in treated group of rats was confirmed after one week of STZ injection (i.e., diabetes inducing period) by estimation of serum glucose level. The rats were fasted for 12 h (free access to water) and treated with D-glucose (2 gm/kg, p.o.). The blood glucose levels were determined before glucose treatment and 120 min after the glucose treatment by using glucometer and glucose testing strips. Rats with blood glucose level ≥ 140 mg/dL at 0 min and ≥ 200 mg/dL at 120 min were considered to be diabetic and included in the study [20–23].

### 2.6. Treatment Schedule

The experimental animals were divided into 5 different groups containing six animals in each group during the drug treatment period. The rats in group I were treated with normal distilled water and served as the normal control. The diabetic rats of treated groups were randomly divided into four groups. Group II diabetic rats were treated with normal distilled water and served as the diabetic control, group III diabetic rats were treated with metformin (10 mg/kg), and diabetic rats of group IV and group V known as test groups were treated with MESG 200 mg/kg and 400 mg/kg, respectively. The dose volume used for each drug was 5 mL/kg in distilled water. Each drug treatment lasted for 4 weeks during which rats of all the groups had free access to normal laboratory food and water. All the drugs were administered in between 9.00 and 10.00 a.m.

### 2.7. Parameters Measured

The different parameters measured during different occasion of experimental period are as follows.

#### 2.7.1. Body Weight, Abdominal Girth, and Food Intake

The body weight and abdominal girth of each animal were recorded on alternate weeks, that is, on days 0, 14, and 28, of both diet manipulating and drug treatment period. The abdominal girth at the level of kidneys was measured as an indicator of abdominal fat [24].

The food intake was monitored periodically by weighing the leftovers for each cage. As per the National Centre for Laboratory Animal Sciences, NIN, Hyderabad, the scale of normal laboratory food diet for each rat is 15–20 gm/day. Each cage had three rats. Accordingly, 75 gm/day/cage of normal laboratory food diet or HFD was given to the respective group of animals. The amount of food leftovers after 24 h for each cage was recorded [25].

#### 2.7.2. Fasting Serum Glucose Estimation

Fasting serum glucose of different group animals (fasted for 12 h) was estimated on 0, 14th, and 28th days of the drug treatment period. A drop of blood was collected from the tail tip of rats for the estimation of blood glucose concentration by using glucometer and glucose testing strips [23].

#### 2.7.3. Analyses of Urine Samples

For estimation of urine volume on 0, 14th, and 28th days of the drug treatment period, the animals were kept in the metabolic cages for 12 h during night period and urine samples were collected. For estimation of urine contents, the rats were placed on clean white ceramic tiles on the morning time, and then their pelvic regions were gently massaged. This treatment induced the release of urine from the fasted animals. Thereafter, the urine was transferred into clean tubes for analyzing the presence of glucose and ketone bodies by using urine analysis reagent strips [26].

#### 2.7.4. Measurement of Insulin Sensitivity

In order to find out the insulin sensitivity of various groups of rats, a simple intravenous insulin tolerance test (IVITT) was carried out on the 25th day of drug treatment period [25, 27]. All the rats were fasted for 3 h before the IVITT. The animals were anesthetized by an i.p. injection of ketamine (70 mg/kg) and received an intravenous injection of short effect human insulin (0.1 U/kg) in the tail veins. Thereafter, blood was collected from the tail tip of the rats for glucose estimation at 0 (before insulin injection), 4, 8, 12, and 16 min of insulin treatment for glucose estimation. The standard curve of blood glucose concentration versus time was plotted. Insulin sensitivity was measured by the glucose disappearance rate, evident from average slope *K* in the fitting curve. The slope of blood glucose disappearance was calculated by linear regression during the period. The respective *K*-value (mg/dL/min) was calculated by multiplying the slope by −1.

#### 2.7.5. Analyses of Serum Samples

For estimation of different biochemical parameters, blood was withdrawn from the retroorbital plexus of the rats (fasted for 12 h) by sterilized capillary tubes under light ether anesthesia on the final day of drug treatment period. The blood was collected in a clean test tube and allowed to coagulate for 30 minutes at room temperature and then centrifuged at 3000 rpm for 15 min. The serum, used as specimen, should be free from hemolysis and hence separated from the clot promptly [28]. The resulting upper serum layer was collected in properly cleaned, dried, and labeled Eppendorf tubes and was stored at 2–8°C for further analysis of different parameters, that is, lipid profile and serum glutamate oxaloacetate transaminase (SGOT) and serum glutamate pyruvate transaminase (SGPT) and serums creatinine, urea, protein, superoxide dismutase (SOD), and malondialdehyde (MDA) [29–32].

#### 2.7.6. Collection of Different Organs

On 28th day of drug treatment period, the animals of different groups were euthanized by cervical dislocation process. The organs, the liver, and pancreas were immediately dissected out and washed in ice-cold saline solution to remove the blood. Extraneous tissues were removed and then pancreas-to-body ratio [20] and hepatic glycogen [33] were estimated.

### 2.8. Statistical Analysis

Differences among treatment group means were assessed by one-way ANOVA (nonparametric), followed by Bonferroni's multiple comparison tests (GraphPad Prism, 5.04 version), and group means were considered to be significantly different at 5% level of significance, *P* < 0.05. The values were expressed as mean ± SEM.

## 3. Results

### 3.1. Phytochemical Screening

The percentage yield (w/w) of MESG was 9.7%. Phytochemical analysis revealed the presence of alkaloids, carbohydrates, proteins, tannins and phenolic compounds, flavonoids, triterpenoids, saponins, steroids, and coumarins in the extract.

### 3.2. Body Weight, Abdominal Girth, and Food Intake

The treated group animals exhibited significantly (*P* < 0.05) higher mean body weight and abdominal girth compared to the diet control group animals at the end of the fourth week of diet manipulating period as well as during diabetes induced period. It was found that the loss of body weight of diabetic control group rats was faster compared to MESG and metformin treated animals during drug treatment period. The percentage reduction of body weight of diabetic control and metformin treated animals was found to be 16% and 7.41%, respectively, on 28th day of drug treatment. Animals treated with 200 and 400 mg/kg of MESG had 6.07% and 4.87% reduction of body weight on the 14th day, respectively, and further gained the body weight by 2.76% and 3.25% on 28th day of drug treatment period, respectively. The metformin and MESG treatment significantly (*P* < 0.05) reduced the abdominal girth during drug treatment period in comparison to diabetic control group (Table 1).

The food intake was monitored periodically by weighing the leftovers for each cage. The food intake was decreased in animals of treated groups fed with HFD by 20–22% at the end of diet manipulating period. Despite decrease in food intake in treated group animals, the total caloric intake was observed to be more in comparison to the normal control group animals. During the drug treatment period, the animals of different groups (i.e., normal control and treated groups) were fed normal laboratory diet. The food intake of diabetic control group animals was more compared to the metformin and MESG treated group animals. Treatment with MESG 200 and 400 mg/kg restored the normal food intake (Table 1).

### 3.3. Fasting Serum Glucose Estimation

After 4 weeks of dietary manipulation and injection of STZ (35 mg/kg, i.p.), significantly (*P* < 0.001) increased serum glucose concentration (approximately 3 fold) in HFD-fed rats compared to normal control rats. Blood glucose remained consistently elevated in diabetic control rats throughout the drug treatment period. Glucose concentration was reduced significantly after oral administration of metformin by 42.88%, MESG 200 mg/kg by 18.76% and MESG 400 mg/kg by 27.49% on 28th day of drug treatment (Table 2).

### 3.4. Analysis of Urine Samples

All the diabetic animals had significantly (*P* < 0.001) increased urine volume compared to the normal control group at day 0 of drug treatment. The metformin and MESG treatment significantly reduced the urine volume on the 28th day of drug treatment. Urine analysis on day 0 of drug treatment revealed the presence of glucose in the urine of all animals, except group I animals. In contrast, ketone bodies were absent in all animals. However, on the 28th day, glucose and ketone bodies were absent in all animals except group II animals (Table 2).

### 3.5. Measurement of Insulin Sensitivity

**Figure 1:**
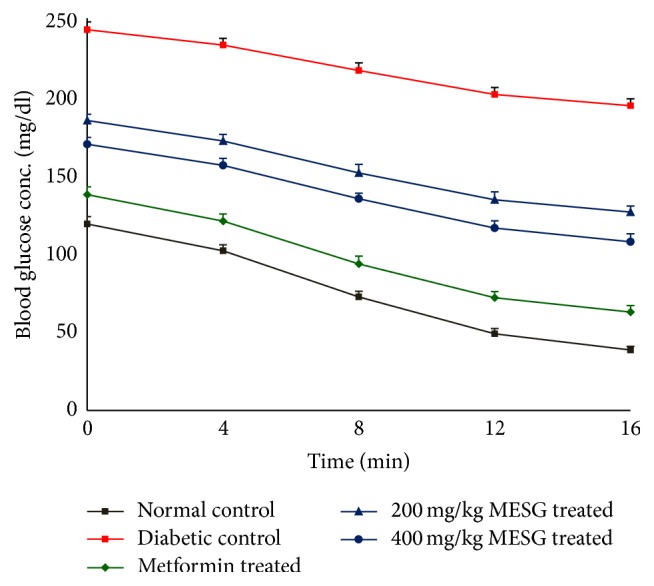
Blood glucose disappearance slope.

On IVITT, it was found that glucose disappearance rate (*K*-value) remained lower in diabetic control rats compared to normal control rats. The *K*-value of diabetic control group was found to be 3.212 and that of normal control group was 5.337. There was increase in *K*-value in the metformin and MESG treated groups. The *K*-value of metformin treated group was found to be 4.962; and MESG 200 and 400 mg/kg treated animals were found to be 3.862 and 4.108, respectively (Table 3, Figure 1).

### 3.6. Analysis of Serum Samples

Level of serum lipid was significantly altered which increased the level of cholesterol, triglycerides, low density lipoprotein (LDL), and very low density lipoprotein (VLDL) and decreased the level of high density lipoprotein (HDL) at the end of the drug treatment period in diabetic control animals as compared to normal control animals. The administration of metformin and MESG to diabetic rats restored the changes in the lipid level to near normal (Table 4).

Serums SGOT and SGPT were significantly increased at the end of drug treatment period in diabetic control animals compared to normal control animals. Metformin treatment significantly (*P* < 0.001) decreased SGOT level (27.88%) and SGPT level (46.92%). The administration of MESG 200 and 400 mg/kg also significantly (*P* < 0.001) decreased SGOT (13.82% and 20.05%) and SGPT level (23.55% and 38.5%), respectively (Table 4).

Serum creatinine and urea were found to be significantly more during end of drug treatment period in diabetic control animals as compared to normal control. Administration of metformin significantly (*P* < 0.001) decreased 54.73% of creatinine level and 44.79% of serum urea level. Administration of MESG 200 and 400 mg/kg reduced creatinine level by 24.92% and 41.94% and serum urea level by 18.72% and 29.15%, respectively (Table 4).

Total protein level was significantly decreased in diabetic control animals (3.47 mg/dL) compared to normal control animals (6.72 mg/dL). The animals treatment with metformin and MESG restored the decreased level of serum total protein to near-normal level. Oral administration of metformin and MESG 200 mg/kg and 400 mg/kg significantly increased the protein level by 82.86, 45.56, and 69.90%, respectively, as compared to diabetic control group (Table 4).

In diabetic control animals, serum SOD level (19.92 U/mL) was significantly decreased and serum MDA level (14.28 nmole/mL) was significantly increased as compared to normal control animals (31.73 U/mL of serum SOD level and 6.23 nmole/mL of serum MDA level). Oral administration of metformin remarkably increased (*P* < 0.001) serum SOD level by 44.77% and decreased (*P* < 0.001) serum MDA level by 46.32%. On administration of MESG 200 mg/kg and 400 mg/kg, the increase in serum SOD level was 29.46% and 36.4% and decrease in serum MDA level was 24.52% and 38.06% (*P* < 0.001), respectively, as compared to diabetic control group (Table 4).

### 3.7. Pancreas-to-Body Ratio

Pancreas-to-body weight ratio (%) of diabetic control rats decreased significantly (0.14%) when compared with that of the normal control rats (0.27%). Pancreas-to-body weight ratio was significantly (*P* < 0.01) increased in the metformin (0.25%) and MESG 200 and 400 mg/kg (0.19% and 0.23%, resp.) treated animals (Table 5).

### 3.8. Hepatic Glycogen Measurement

Hepatic glycogen level was reduced significantly in diabetic control animals (6.03 mg/dL) as compared to normal control animals (14.61 mg/dL). The diabetic animals treated with metformin and MESG showed high concentration of hepatic glycogen as compared to diabetic control group. The increase in hepatic glycogen level was found to be 105.3, 65.5, and 89.85% in metformin, MESG 200 mg/kg, and MESG 400 mg/kg treatment groups, respectively, as compared to diabetic control group (Table 5).

## 4. Discussion

Type 2 diabetes is chronic and progressive disease which may be the consequence of impaired insulin secretion by *β*-cells of pancreas, resistance of peripheral tissue to insulin action, and augmented hepatic glucose production [34, 35]. Natural remedies have been greatly explored for the management of diabetes due to their low side effects as compared to the conventional therapies. The present study elucidated the antidiabetic efficacy of MESG by determining a number of parameters of biological significance.

The body weight was measured as an indicator of growth, and abdominal girth at the level of kidneys was measured as an indicator of abdominal fat. Greater consumption of high energy content foods such as HFD leads to an increase in the fat mass (adiposity) and fat cell enlargement (hypertrophy), producing the characteristic pathology of obesity [36]. Adipose tissue plays a major role in regulating whole body insulin resistance. The increase in the prevalence of obesity has been accompanied by a parallel increase in the prevalence of T2DM [29]. The body weight of diabetic control group was decreased faster during the treatment period in our study. The characteristic loss of body weight is caused due to an increase muscle wasting in diabetes. Metformin treatment reduced the body weight, but slowly during the drug treatment period as compared to diabetic control animals. MESG 200 and 400 mg/kg reduced the body weight (slower than other groups) during 14th day of drug treatment period and then animals gained the body weight during 28th day. The mechanism of this extract may be similar to that of sulfonylureas, because treatment with sulfonylureas is associated with weight gain [37]. This data confirms our previous findings; that is, MESG treatment decreases the serum glucose level, as that of sulfonylurea, in glucose loaded hyperglycemic rats and hypoglycemic activity in normal rats [38].

Due to decreased sensitivity of insulin receptors in T2DM, insulin catabolism of protein and fats occurs resulting in removal of gluconeogenic amino acid from the liver, which results in negative energy balance which in turn leads to increasing appetite, that is, polyphagia [39]. In the present study, food intake of diabetic control group was increased during drug treatment period. Metformin and MESG treatment significantly restored the food intake to normal level. Hyperglycemia has an important role in the pathogenesis of long-term complications during diabetes. Oral administration of metformin and MESG has significantly reduced glucose concentration.

In T2DM, because of insulin deficiency, glucose assimilation in the muscle and liver is greatly reduced and also the stores of glycogen are depleted by increased glycogenolysis. This causes glycosuria and induces osmosis, thus resulting in polyuria [39]. During drug treatment period, diabetic control animals had significantly (*P* < 0.001) higher mean urine volume compared to the normal control groups, but metformin and MESG treatment significantly reduced the urine volume.

Insulin deficiency also stimulates the release of epinephrine that enhances the release of glucagons. This disturbed insulin-to-glucagon ratio stimulates lipoprotein lipase, with the resultant breakdown of adipose stores and an increase in the levels of free fatty acids (FFA). FFA reaches the liver and gets esterified to fatty acyl CoA. Oxidation of this fatty acyl CoA within the mitochondria produces ketone bodies such as acetoacetic acid and *β*-hydroxybutyric acid. Rate of formation of these ketone bodies exceeds their utilization in peripheral tissues causing ketonemia and ketonuria [39]. On day 0 of drug treatment period, glucose was found in the urine of all diabetic rats and the ketone bodies were absent in all rats. At the end of drug treatment period, the glucose and ketone bodies were found in the urine of diabetic control group rats while being absent in metformin and MESG treated animals.

*K*-values were measured by the glucose disappearance rate, evident from average slope *K* in the fitting curve, and are used to describe excess of insulin sensitivity. Higher *K*-value suggests faster serum glucose disappearance and higher insulin sensitivity after injecting exogenous insulin. Hence, this model with the involvement of both insulin resistance and obvious *β*-cell dysfunction in the development of diabetes could be suitable for studying the pathophysiology of type 2 diabetes as well as for testing new compounds, which act through ameliorating insulin resistance and/or by increasing *β*-cell insulin secretion [27]. The frank hyperglycemia in the presence of comparable amount of plasma insulin concentrations together with reduced *K*-value indicated the persistence of insulin resistance in HFD-fed and STZ treated diabetic control rats. Our study showed a significant increase in *K*-value in the metformin and MESG treated group animals.

Under normal circumstances, insulin activates the enzyme lipoprotein lipase, which hydrolyses triglycerides. However, in diabetic state, lipoprotein lipase is not activated due to insulin deficiency, resulting in hypertriglyceridemia and hypercholesterolemia (also due to metabolic abnormalities) [29]. Serum lipid level was significantly altered in diabetic control animals as compared to normal control animals during end of the drug treatment period, whereas administration of metformin and MESG restored the changes to near normal.

Elevated levels of SGOT and SGPT are found in different disease conditions including diabetic state [25] and serve as the pathophysiological markers. In our study the serum SGOT and SGPT levels were significantly high in diabetic control animals as compared to normal control animals during the end of drug treatment period. The administration of metformin and MESG significantly decreased the increased enzyme levels. The improvements in the levels of liver enzymes in the diabetic animals could be beneficial in preventing diabetic complications, as well as improving lipid and protein metabolism in diabetic liver.

In diabetes mellitus, the amino acid breakdown in the liver results in an increased production of urea and creatinine. In diabetic state, there is increased metabolism of proteins and amino acids and this leads to decrease in serum protein level [30]. Serum creatinine and urea levels were increased significantly and serum protein level was significantly decreased in diabetic control animals compared to normal control animals. The treatment of metformin and MESG significantly decreased the serum creatinine and urea level and increased the serum protein level to near-normal levels.

The SOD is an important defense enzyme which neutralizes the effect of superoxide anion during the oxidative stress in the tissues. Oxidative stress generally causes damage to the membrane polyunsaturated fatty acids (PUFA) leading to generation of malondialdehyde (MDA), a thiobarbituric acid reacting substance (TBARS). Several studies have indicated an increase in serum TBARS and a decrease in plasma SOD activity signifying an imbalance between the prooxidant and antioxidant states in the body, leading to an imbalance in systemic redox status [40]. In the present study, we found significant decrease in serum SOD and elevated MDA content activity in diabetic control animals as compared to normal control animals, signifying an imbalance between the prooxidant and antioxidant states. The animals treatment with metformin and MESG significantly increased the serum SOD level, decreasing level of serum MDA and balancing the prooxidant and antioxidant states in the body.

Pancreas-to-body weight ratio of diabetic control rats decreased significantly when compared with normal control rats. Administration of metformin and MESG to diabetic rats significantly increased the pancreas-to-body weight ratio. This indicates the protective activity of the extract on the pancreas.

Glycogen synthesis in the liver and skeletal muscle is impaired in diabetes [33]. The administration of metformin and MESG significantly increased the decreased hepatic glycogen level in the diabetic animals. The significant increase in the glycogen levels in treated groups may be attributed to the reactivation of the glycogen synthesis system.

The results of the present work provide evidence that MESG can alleviate hyperglycemia, hyperlipemia, and insulin resistance in high fat diet and low dose STZ induced type 2 diabetic rats. MESG exhibited antidiabetic potential in a dose dependent manner, judged by its ability to fully restore the raised blood glucose levels and other parameters in the treated diabetic rats to the normal values by its faster effects. The phytochemical study showed that the extract contains phytochemicals like alkaloids, phenolics, tannins, flavonoids, triterpenoids, and sterols. Our earlier study revealed the total phenolic and flavonoid content and* in vitro* antioxidant potential of MESG [13] which may be responsible for potent hypoglycemic and hypolipidemic properties.

The MESG exerts its hypoglycemic actions through its active phytoconstituents which may be due to either stimulating the release of insulin from the *β*-cells of the pancreas of normal and mildly diabetic animals (pancreatic mechanism) or insulin-mimetic effects, for example, stimulation of cellular processes that consume glucose (extrapancreatic mechanism), or both. On the basis of the outcomes of the present study, it is concluded that* Sesbania grandiflora* has potential antidiabetic activities and the result scientifically justifies their use in the folklore remedies. However, further studies are required to identify the bioactive compounds responsible for the antidiabetic property of the plant species.

## Figures and Tables

**Table 1:** Effect of MESG on body weight, abdominal girth, and food intake during different days of diet manipulating period and drug treatment period.

Treatment group	Parameters	Different days of diet manipulating period			Different days of drug treatment period		
		0	14	28	0	14	28
Group I Normal control	Body weight (gm)	151.67 ± 3.00	160.83 ± 2.39	172.50 ± 3.35	178.33 ± 2.47	187.50 ± 2.14	196.67 ± 3.33
	Abdominal girth (cm)	13.82 ± 0.15	14.28 ± 0.10	14.73 ± 0.20	14.95 ± 0.21	15.35 ± 0.19	15.73 ± 0.15
	Food intake (gm)	15.63 ± 0.47	15.98 ± 0.62	16.45 ± 0.58	16.85 ± 0.88	17.02 ± 0.95	17.18 ± 0.92

Group II Diabetic control	Body weight (gm)	153.33 ± 3.07	182.50 ± 3.10^a^	221.67 ± 3.57^a^	239.17 ± 3.96^a^	224.17 ± 3.00^a^	211.67 ± 2.47^a^
	Abdominal girth (cm)	13.52 ± 0.23	15.62 ± 0.32^c^	17.32 ± 0.19^b^	18.17 ± 0.15^a^	18.38 ± 0.19^a^	18.37 ± 0.19^a^
	Food intake (gm)	17.03 ± 0.80	14.18 ± 0.62	13.52 ± 1.05^a^	14.43 ± 0.60^a^	18.62 ± 0.65^c^	20.23 ± 0.63^b^

Group III Metformin treated	Body weight (gm)	157.50 ± 3.82	186.67 ± 3.33	225.83 ± 3.33	243.33 ± 3.57	235.83 ± 2.39^*∗*^	231.67 ± 3.33^*∗∗∗*^
	Abdominal girth (cm)	12.82 ± 0.36	14.98 ± 0.29	16.65 ± 0.22	17.57 ± 0.20	17.08 ± 0.19^*∗*^	16.52 ± 0.25^*∗∗*^
	Food intake (gm)	16.70 ± 0.87	14.03 ± 0.80	13.48 ± 0.92	14.27 ± 0.77	16.90 ± 0.80	17.18 ± 0.98^*∗*^

Group IV 200 mg/kg MESG treated	Body weight (gm)	150.83 ± 3.07	181.67 ± 2.47^#^	216.67 ± 2.47^#^	235.83 ± 2.39^#^	226.17 ± 3.00^#^	230.83 ± 2.39^*∗∗*#^
	Abdominal girth (cm)	13.10 ± 0.16	15.32 ± 0.12	17.02 ± 0.12	17.93 ± 0.18	17.58 ± 0.19^*∗*^	17.22 ± 0.18^*∗*^
	Food intake (gm)	16.13 ± 0.73	14.48 ± 0.75	12.98 ± 1.08	14.98 ± 1.25	17.05 ± 0.82	17.95 ± 0.98^*∗*^

Group V 400 mg/kg MESG treated	Body weight (gm)	154.17 ± 2.39	187.50 ± 2.81^#^	223.33 ± 3.07^#^	244.17 ± 2.39^#^	236.67 ± 2.47^*∗*#^	241.67 ± 2.47^*∗∗∗*#^
	Abdominal girth (cm)	13.93 ± 0.41	15.77 ± 0.15	17.55 ± 0.20	18.43 ± 0.17	17.95 ± 0.19^*∗*^	17.50 ± 0.19^*∗*^
	Food intake (gm)	15.78 ± 0.38	13.45 ± 0.62	12.40 ± 0.53	13.78 ± 0.65	16.28 ± 1.12	17.43 ± 0.70^*∗*^

The results were expressed as mean ± SEM, *n* = 6.

^a^ *P* < 0.001, ^b^ *P* < 0.01, and ^c^ *P* < 0.05, diabetic control versus normal control.

^*∗∗∗*^ *P* < 0.001, ^*∗∗*^ *P* < 0.01, and ^*∗*^ *P* < 0.05, metformin treated and MESG treated groups versus diabetic control group.

“#” indicates that there is no significant difference between metformin treated and MESG treated groups at *P* < 0.05.

**Table 2:** Effect of MESG on blood glucose level and urine analysis during different days of drug treatment period.

Parameters	Different days	Group I Normal control	Group II Diabetic control	Group III Metformin treated	Group IV 200 mg/kg MESG treated	Group V 400 mg/kg MESG treated
Blood glucose	0	84.67 ± 3.52	239.17 ± 6.11^a^	237.50 ± 5.21	244.17 ± 6.64^#^	243.33 ± 4.91^#^
	14	81.83 ± 2.95	223.33 ± 5.12^a^	162.83 ± 5.21^*∗∗∗*^	201.17 ± 4.53^*∗*^	182.67 ± 4.38^*∗∗∗*#^
	28	83.33 ± 3.15	217.67 ± 5.47^a^	124.33 ± 4.81^*∗∗∗*^	166.50 ± 4.32^*∗∗∗*^	145.17 ± 4.51^*∗∗∗*#^

Urine volume (mL)	0	5.48 ± 0.25	8.32 ± 0.18^a^	8.23 ± 0.22	7.68 ± 0.20^#^	7.82 ± 0.19^#^
	14	5.57 ± 0.22	8.17 ± 0.20^a^	6.02 ± 0.26^*∗∗∗*^	6.67 ± 0.25^*∗∗*#^	6.42 ± 0.25^*∗∗∗*#^
	28	5.28 ± 0.24	8.47 ± 0.21^a^	5.47 ± 0.27^*∗∗∗*^	6.05 ± 0.26^*∗∗∗*#^	5.38 ± 0.29^*∗∗∗*#^

Presence of glucose in urine	0	−	+	+	+	+
	14	−	+	−	−	−
	28	−	+	−	−	−

Presence of ketone bodies in urine	0	−	−	−	−	−
	14	−	−	−	−	−
	28	−	+	−	−	−

The results were expressed as mean ± SEM, *n* = 6.

^a^ *P* < 0.001; diabetic control versus normal control.

^*∗∗∗*^ *P* < 0.001, ^*∗∗*^ *P* < 0.01, and ^*∗*^ *P* < 0.05, metformin treated and MESG treated groups versus diabetic control group.

“#” indicates that there is no significant difference between metformin treated and MESG treated groups at *P* < 0.05.

“+” indicates being present and “−” indicates being absent.

**Table 3:** *K*-values (mg/dL/min) of different groups.

Treatment groups	Linear regression equation for the slope	*K*-value (mg/dL/min)
Group I (normal control)	*y* = −5.337*x* + 118.7	5.337
	*R* ^2^ = 0.980	

Group II (diabetic control)	*y* = −3.212*x* + 243.2	3.212
	*R* ^2^ = 0.985	

Group III (metformin treated)	*y* = −4.962*x* + 136.9	4.962
	*R* ^2^ = 0.978	

Group IV (MESG treated, 200 mg/kg)	*y* = −3.862*x* + 184.5	3.862
	*R* ^2^ = 0.983	

Group V (MESG treated, 400 mg/kg)	*y* = −4.108*x* + 169.7	4.108
	*R* ^2^ = 0.984	

**Table 4:** Effect of MESG on serum parameters during end of the drug treatment period.

Serum parameters	Group I Normal control	Group II Diabetic control	Group III Metformin treated	Group IV 200 mg/kg MESG treated	Group V 400 mg/kg MESG treated
Total cholesterol	59.33 ± 4.03	166.50 ± 5.01^a^	82.67 ± 4.57^*∗∗∗*^	122.17 ± 5.53^*∗∗∗*^	102.17 ± 4.83^*∗∗∗*#^
Triglycerides	53.83 ± 3.93	147.67 ± 5.16^a^	71.50 ± 3.55^*∗∗∗*^	114.83 ± 3.10^*∗∗∗*^	88.83 ± 3.74^*∗∗∗*#^
HDL	29.17 ± 1.66	16.50 ± 1.63^a^	27.67 ± 2.11^*∗∗*^	22.17 ± 1.85	24.33 ± 2.20^*∗*^
LDL	19.40 ± 4.07	120.47 ± 7.02^a^	40.70 ± 4.91^*∗∗∗*^	77.03 ± 6.29^*∗∗∗*^	60.07 ± 4.33^*∗∗∗*#^
VLDL	10.77 ± 0.79	29.53 ± 1.03^a^	14.30 ± 0.71^*∗∗∗*^	22.97 ± 0.62^*∗∗∗*^	17.77 ± 0.75^*∗∗∗*#^
SGOT (U/L)	41.17 ± 1.30	72.33 ± 2.51^a^	52.17 ± 1.87^*∗∗∗*^	62.33 ± 2.17^*∗*^	57.83 ± 2.61^*∗∗∗*#^
SGPT (U/L)	38.83 ± 2.65	89.17 ± 2.69^a^	47.33 ± 2.94^*∗∗∗*^	68.17 ± 3.71^*∗∗∗*^	54.83 ± 3.21^*∗∗∗*#^
Creatinine (mg/dL)	0.54 ± 0.06	1.50 ± 0.07^a^	0.68 ± 0.05^*∗∗∗*^	1.13 ± 0.08^*∗∗*^	0.87 ± 0.07^*∗∗∗*#^
Serum urea (mg/dL)	33.17 ± 2.09	70.33 ± 3.39^a^	38.83 ± 3.10^*∗∗∗*^	57.17 ± 3.56^*∗*^	49.83 ± 3.74^*∗∗*#^
Serum protein (gm/dL)	6.72 ± 0.32	3.47 ± 0.30^a^	6.35 ± 0.37^*∗∗∗*^	5.05 ± 0.37^*∗*#^	5.90 ± 0.33^*∗∗∗*#^
Serum SOD (U/mL)	31.73 ± 1.18	19.92 ± 1.26^a^	28.83 ± 1.10^*∗∗∗*^	25.78 ± 1.24^*∗*#^	27.17 ± 1.31^*∗∗*#^
Serum MDA (nmole/mL)	6.23 ± 0.62	14.28 ± 1.04^a^	7.67 ± 0.85^*∗∗∗*^	10.78 ± 0.70^*∗*#^	8.85 ± 0.76^*∗∗*#^

The results were expressed as mean ± SEM, *n* = 6.

^a^ *P* < 0.001; diabetic control versus normal control.

^*∗∗∗*^ *P* < 0.001, ^*∗∗*^ *P* < 0.01, and ^*∗*^ *P* < 0.05, metformin treated and MESG treated groups versus diabetic control group.

“#” indicates that there is no significant difference between metformin treated and MESG treated groups at *P* < 0.05.

**Table 5:** Pancreas-to-body weight ratio and hepatic glycogen content of different treatment groups.

Treatment group	Pancreas-to-body weight ratio (%)	Hepatic glycogen (mg/gm)
Group I Normal control	0.27 ± 0.02	14.61 ± 0.79

Group II Diabetic control	0.14 ± 0.04^a^	6.03 ± 0.72^a^

Group III Metformin treated	0.25 ± 0.03^*∗∗*^	12.38 ± 0.73^*∗∗∗*^

Group IV 200 mg/kg MESG treated	0.19 ± 0.01^#^	9.98 ± 0.72^*∗*#^

Group V 400 mg/kg MESG treated	0.23 ± 0.02^*∗*#^	11.45 ± 0.87^*∗∗∗*#^

The results were expressed as mean ± SEM, *n* = 6.

^a^ *P* < 0.001; diabetic control versus normal control.

^*∗∗∗*^ *P* < 0.001, ^*∗∗*^ *P* < 0.01, and ^*∗*^ *P* < 0.05, metformin treated and MESG treated groups versus diabetic control group.

“#” indicates that there is no significant difference between metformin treated and MESG treated groups at *P* < 0.05.

## References

[1] Ballington D. A., Laughlin M. M. (2006). *Pharmacology*.

[2] Rahimi R., Nikfar S., Larijani B., Abdollahi M. (2005). A review on the role of antioxidants in the management of diabetes and its complications. *Biomedicine and Pharmacotherapy*.

[3] Wild S., Roglic G., Green A., Sicree R., King H. (2004). Global prevalence of diabetes estimates for the year 2000 and projections for 2030. *Diabetes Care*.

[4] Shaw J. E., Sicree R. A., Zimmet P. Z. (2010). Global estimates of the prevalence of diabetes for 2010 and 2030. *Diabetes Research and Clinical Practice*.

[5] Whiting D. R., Guariguata L., Weil C., Shaw J. E. (2011). IDF diabetes atlas: global estimates of the prevalence of diabetes for 2011 and 2030. *Diabetes Research and Clinical Practice*.

[6] Kasture V. S., Deshmukh V. K., Chopde C. T. (2002). Anxiolytic and anticonvulsive activity of *Sesbania grandiflora* leaves in experimental animals. *Phytotherapy Research*.

[7] Laohabutr P., Kangsadalampai O., Panmaung T., Tongyonk L. (2001). Iron, Vitamin C, phytate and crude fiber contents in northeastern local vegetables. *Thai Journal of Pharmaceutical Sciences*.

[8] Kalyanagurunathan P., Sulochana N., Murugesh N. (1985). *In vitro* haemolytic effect of the flowers of *Sesbania grandiflora*. *Fitoterapia*.

[9] Fojas F. R., Barrientos C. M., Capal T. V. (1982). Preliminary phytochemical and pharmacological studies of *Sesbania grandiflora* (l.) Pers. *Philippine Journal of Science*.

[10] Mokkhasmit M., Ngarmwathana W., Sawasdimongkol K., Permphiphat U. (1971). Pharmacological evaluation of Thai medicinal plants. *Journal of the Medical Association of Thailand*.

[11] Khan M. A., Khan T., Ahmad Z. (1994). Barks used as source of medicine in Madhya Pradesh, India. *Fitoterapia*.

[12] Mackeen M. M., Ali A. M., El-Sharkawy S. H. (1997). Antimicrobial and cytotoxic properties of some Malaysian traditional vegetables (Ulam). *International Journal of Pharmacognosy*.

[13] Panda C., Mishra U. S., Mahapatra S., Panigrahi G. (2013). Free radical scavenging activity and phenolic content estimation of *Glinus oppositifolius* and *Sesbania grandiflora*. *International Journal of Pharmacy*.

[14] Kokate C. K., Purohit A. P., Gokhale S. B. (2002). *Pharmacognosy*.

[15] Evans W. C. (2002). *Trease and Evans Pharmacognosy*.

[16] Khandelwal K. R. (2005). *Practical Pharmacognosy*.

[17] Kraegen E. W., Clark P. W., Jenkins A. B., Daley E. A., Chisholm D. J., Storlien L. H. (1991). Development of muscle insulin resistance after liver insulin resistance in high-fat-fed rats. *Diabetes*.

[18] Storlien L. H., James D. E., Burleigh K. M., Chisholm D. J., Kraegen E. W. (1986). Fat feeding causes widespread *in vivo* insulin resistance, decreased energy expenditure, and obesity in rats. *American Journal of Physiology Endocrinology and Metabolism*.

[19] Reed M. J., Meszaros K., Entes L. J. (2000). A new rat model of type 2 diabetes: the fat-fed, streptozotocin-treated rat. *Metabolism: Clinical and Experimental*.

[20] Zhang J., Huang Y. L., Hou T. D., Wang Y. P. (2006). Hypoglycaemic effect of *Artemisia sphaerocephala* Krasch. Seed polysaccharide in alloxan-induced diabetic rats. *Swiss Medical Weekly*.

[21] Srinivasan K., Viswanad B., Asrat L., Kaul C. L., Ramarao P. (2005). Combination of high-fat diet-fed and low-dose streptozotocin-treated rat: a model for type 2 diabetes and pharmacological screening. *Pharmacological Research*.

[22] Sugano M., Yamato H., Hayashi T. (2006). High-fat diet in low-dose-streptozotocin-treated heminephrectomized rats induces all features of human type 2 diabetic nephropathy: a new rat model of diabetic nephropathy. *Nutrition, Metabolism and Cardiovascular Diseases*.

[23] Xing X.-H., Zhang Z.-M., Hu X.-Z., Wu R.-Q., Xu C. (2009). Antidiabetic effects of *Artemisia sphaerocephala* Krasch. gum, a novel food additive in China, on streptozotocin-induced type 2 diabetic rats. *Journal of Ethnopharmacology*.

[24] Modak T., Mukhopadhaya A. (2011). Effects of citral, a naturally occurring antiadipogenic molecule, on an energy-intense diet model of obesity. *Indian Journal of Pharmacology*.

[25] Xie W., Nie Y., Du L., Zhang Y., Cai G. (2007). Preventive effects of fenofibrate on insulin resistance, hyperglycaemia, visceral fat accumulation in NIH mice induced by small-dose streptozotocin and lard. *Pharmacological Research*.

[26] Onoagbe I. O., Negbenebor E. O., Ogbeide V. O. (2010). A study of the anti-diabetic effects of *Urena lobata* and *Sphenostylis stenocar*pa in streptozotocin-induced diabetic rats. *European Journal of Scientific Research*.

[27] Gelding S. V., Robinson S., Lowe S., Niththyananthan R., Johnston D. G. (1994). Validation of the low dose short insulin tolerance test for evaluation of insulin sensitivity. *Clinical Endocrinology*.

[28] Parasuraman S., Raveendran R., Kesavan R. (2010). Blood sample collection in small laboratory animals. *Journal of Pharmacology and Pharmacotherapeutics*.

[29] Shah S. S., Shah G. B., Singh S. D. (2011). Effect of piperine in the regulation of obesity-induced dyslipidemia in high-fat diet rats. *Indian Journal of Pharmacology*.

[30] Verma L., Khatri A., Kaushik B., Patil U. K., Pawar R. S. (2010). Antidiabetic activity of *Cassia occidentalis* (Linn) in normal and alloxan-induced diabetic rats. *Indian Journal of Pharmacology*.

[31] Kakkar P., Das B., Viswanathan P. N. (1984). A modified spectrophotometric assay of superoxide dismutase. *Indian Journal of Biochemistry and Biophysics*.

[32] Dahle L. K., Hill E. G., Holman R. T. (1962). The thiobarbituric acid reaction and the autoxidations of polyunsaturated fatty acid methyl esters. *Archives of Biochemistry and Biophysics*.

[33] Musabayane C. T., Mahlalela N., Shode F. O., Ojewole J. A. O. (2005). Effects of *Syzygium cordatum* (Hochst.) [Myrtaceae] leaf extract on plasma glucose and hepatic glycogen in streptozotocin-induced diabetic rats. *Journal of Ethnopharmacology*.

[34] Shulman G. I. (2000). Cellular mechanisms of insulin resistance. *The Journal of Clinical Investigation*.

[35] Muhammed S. J., Lundquist I., Salehi A. (2012). Pancreatic *β*-cell dysfunction, expression of iNOS and the effect of phosphodiesterase inhibitors in human pancreatic islets of type 2 diabetes. *Diabetes, Obesity and Metabolism*.

[36] Fontaine K. R., Redden D. T., Wang C., Westfall A. O., Allison D. B. (2003). Years of life lost due to obesity. *The Journal of the American Medical Association*.

[37] Bailey C. J. (2011). Renal glucose reabsorption inhibitors to treat diabetes. *Trends in Pharmacological Sciences*.

[38] Panigrahi G., Panda C., Mishra U. S., Mahapatra S., Pasa G., Acharya A. K. (2012). Investigation of possible hypoglycemic and hypolipidemic effect of methanolic extract of *Sesbania grandiflora*. *International Research Journal of Pharmacy*.

[39] Kumar V., Abbas A. K., Fausto N. (2005). *Robbins and Cottran Pathologic Basis of Disease*.

[40] Klug-Roth D., Fridovich I., Rabani J. (1973). Pulse radiolytic investigations of superoxide catalyzed disproportionation. Mechanism for bovine superoxide dismutase. *Journal of the American Chemical Society*.

